# A role for steroid 5 alpha-reductase 1 in vascular remodeling during endometrial decidualization

**DOI:** 10.3389/fendo.2022.1027164

**Published:** 2022-11-16

**Authors:** Isaac W. Shaw, Phoebe M. Kirkwood, Diane Rebourcet, Fiona L. Cousins, Rebecca J. Ainslie, Dawn E. W. Livingstone, Lee B. Smith, Philippa T.K. Saunders, Douglas A. Gibson

**Affiliations:** ^1^ Centre for Inflammation Research, The University of Edinburgh, Edinburgh, United Kingdom; ^2^ MRC Centre for Reproductive Health, The University of Edinburgh, Edinburgh, United Kingdom; ^3^ Centre for Discovery Brain Science, The University of Edinburgh, Edinburgh, United Kingdom

**Keywords:** decidualization, angiogenesis, dihydrotestosterone, 5 alpha-reductase, androgen, intracrinology, vascular remodeling, finasteride

## Abstract

Decidualization is the hormone-dependent process of endometrial remodeling that is essential for fertility and reproductive health. It is characterized by dynamic changes in the endometrial stromal compartment including differentiation of fibroblasts, immune cell trafficking and vascular remodeling. Deficits in decidualization are implicated in disorders of pregnancy such as implantation failure, intra-uterine growth restriction, and pre-eclampsia. Androgens are key regulators of decidualization that promote optimal differentiation of stromal fibroblasts and activation of downstream signaling pathways required for endometrial remodeling. We have shown that androgen biosynthesis, *via* 5α-reductase-dependent production of dihydrotestosterone, is required for optimal decidualization of human stromal fibroblasts *in vitro*, but whether this is required for decidualization *in vivo* has not been tested. In the current study we used steroid 5α-reductase type 1 (SRD5A1) deficient mice (*Srd5a1-/-* mice) and a validated model of induced decidualization to investigate the role of SRD5A1 and intracrine androgen signaling in endometrial decidualization. We measured decidualization response (weight/proportion), transcriptomic changes, and morphological and functional parameters of vascular development. These investigations revealed a striking effect of 5α-reductase deficiency on the decidualization response. Furthermore, vessel permeability and transcriptional regulation of angiogenesis signaling pathways, particularly those that involved vascular endothelial growth factor (VEGF), were disrupted in the absence of 5α-reductase. In *Srd5a1-/-* mice, injection of dihydrotestosterone co-incident with decidualization restored decidualization responses, vessel permeability, and expression of angiogenesis genes to wild type levels. Androgen availability declines with age which may contribute to age-related risk of pregnancy disorders. These findings show that intracrine androgen signaling is required for optimal decidualization *in vivo* and confirm a major role for androgens in the development of the vasculature during decidualization through regulation of the VEGF pathway. These findings highlight new opportunities for improving age-related deficits in fertility and pregnancy health by targeting androgen-dependent signaling in the endometrium.

## Introduction

Decidualization is a fundamental step in the establishment of pregnancy that involves coordinated remodeling of the endometrial stroma. It is a hormone-dependent process characterized by differentiation of endometrial stromal fibroblasts (hESF) (1), immune cell trafficking (2) and vascular remodeling (3). Deficits in decidualization are implicated in disorders of pregnancy such as implantation failure, intra-uterine growth restriction, and pre-eclampsia (4, 5). Risk of pregnancy disorders increases with age which may be associated with age-related decline in hormone production or availability.

Androgens are key regulators of decidualization that promote optimal differentiation of stromal fibroblasts and activation of downstream signaling pathways required for endometrial remodeling. Androgen supplementation enhances secretion of decidualization markers in hESF *in vitro* (6). Exogenous dihydrotestosterone (DHT) enhances and maintains decidualization responses in mice, an effect which is attenuated by co-administration of the AR antagonist flutamide (7). Endometrial fibroblasts in both mouse and human endometrium express androgen receptor (AR) and their function is altered by AR-dependent signaling (8–10). Using assays that combined *in vitro* decidualization and knockdown of receptors Cloke et al. reported that AR regulated distinct decidual gene networks involved in cytoskeletal organization, cell motility and regulation of the cell cycle (11). Using a pharmacologic approach, we found that flutamide attenuated the expression of decidualization and endometrial receptivity markers in hESF (12). Together, these studies confirm the importance of androgens and AR-dependent signaling for optimal decidualization responses.

In women, androgens and their precursors are abundant in the circulation but production declines with age (13, 14). Circulating concentrations of the most potent androgen dihydrotestosterone (DHT) are low but this is because DHT is primarily a product of local metabolism within target tissues in women; mediating its affects *via* intracrine signaling (15, 16). We have shown that DHT is actively produced by endometrial stromal fibroblasts during decidualization *via* expression of the enzyme SRD5A1 and that the abundance of DHT is affected by availability of steroid precursors such as dehydroepiandrosterone (DHEA) (5, 12, 17, 18). When we increased intracrine DHT signaling by supplementing with DHEA we found that decidualization responses were enhanced and expression of implantation markers were also increased. Androgen precursor availability and intracrine androgen signaling can therefore dictate the extent of decidualization responses.

To date, a role for intracrine androgen signaling in regulating decidualization *in vivo* has not been rigorously investigated. Previous studies have been hampered by the developmental uterine defects reported in global AR knock-out female mice (19). In the current study, we have used mice which are homozygous for a recessive knock-out mutation in the gene encoding the type 1 steroid 5α-reductase (SRD5A1) enzyme (*Srd5a1^-/-^*) which lack the capacity to convert T to DHT (20). Previously, Srd5a1^-/-^ mice have been shown to have reduced fertility due to estrogen-induced fetal death and a parturition defect caused by lack of 5α-androstan-3α,17β-diol, another product of SRD5A1 (21). The potential role of this enzyme and the contribution of 5α-reduced androgens to decidualization has not been tested, but it is plausible that the reduced fecundity reported in Srd5a1^-/-^ mice (22) may in part be due to decidualization defects as a result of intracrine androgen deficiency. To investigate this, we have assessed the impact of 5α-reductase deletion or inhibition in a mouse model of induced decidualization.

We show that 5α-reductase deficiency leads to impaired decidualization, structural and functional changes to decidual blood vessels, and transcriptomic changes affecting angiogenesis signaling pathways. Deficits in decidualization in Srd5a1^-/-^ mice were reversed by restoration of androgen signaling through exogenous DHT administration. We conclude that intracrine androgens are necessary for optimal decidualization and vascular remodeling required for establishment of pregnancy.

## Materials and methods

### Mice

All mouse work was performed in accordance with the Animals (Scientific Procedures) Act 1986 under UK law and were bred on a C57Bl/6J or C57Bl6/JCrl background. The Srd5a1^-/-^ mouse line was obtained from the Jackson Laboratories (https://www.jax.org/strain/002793). The original paper describing the generation of mice with a null allele was published by Mahendroo, Cala, and Russel (20). Due to parturition defects in homozygous mice heterozygote crosses were used to maintain the colony and produce *Srd5a1^-/-^* progeny. Wildtype (WT) mice were either littermates or purchased from Charles River Laboratories (Tranent, Scotland). Expression of 5α-Reductase isozymes (types 1-3) was assessed in uterine tissues by qPCR (**Supplementary Figures 1**). *Srd5a1* was absent in uterine tissues from knockout mice, and consistent with previous reports (20), *Srd5a2* was not detected in either wildtype or knockout mice. While *Srd5a3* was detected its expression was decreased with decidualization (**Supplementary Figures 1**).

### Mouse model of induced decidualization

**Figure 1:**
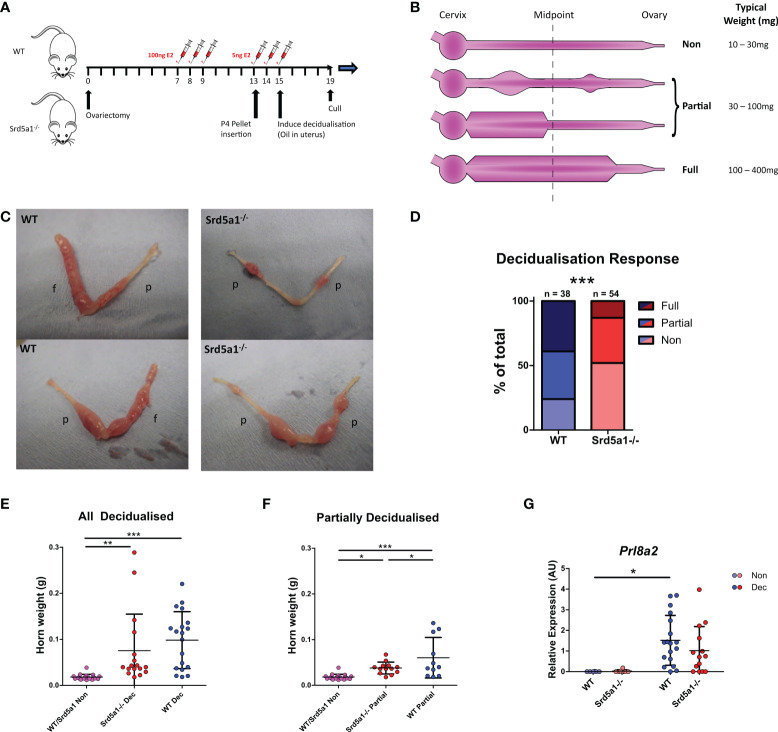
Decidualization response is impaired in Srd5a1^-/-^ mice. **(A)** Schema for the decidualization induction model. E2 = estradiol. P4 = progesterone. **(B)** Graphic depicting a uterine horn in various stages of decidualization. The horn is thicker where a decidualization reaction has occurred. A horn is deemed fully decidualized if an uninterrupted stretch of decidualization has occurred over at least half of the horn length. Partial decidualization is any decidualization response less than this, often occurring as disparate nodes along the horn. **(C)** Representative images of uterine horns from wild type (WT) and Srd5a1^-/-^ animals, with the classification of each horn given; p = partial decidualization, f = fully decidualized. **(D)** Contingency table depicting proportion of horn responses in each genotype. **(E, F)** Wet weight of uterine horns in WT and Srd5a1^-/-^ mice at time of tissue recovery (4 days following decidualization induction). Non-decidualized horns have been pooled. E compares all horns with a decidualization reaction (Full & Partial), whereas F shows analysis of those with a partial reaction. **(G)** Expression of *Prl8a2 via* qPCR in uterine horns of WT and Srd5a1^-^/^-^ mice following decidualization induction. Plots in D analyzed by chi-squared test for trend; **(E, F)** analyzed by one-way ANOVA with Newman-Keuls *post-hoc* tests; and G analyzed by two-way ANOVA with Bonferroni *post-hoc* tests; ***p < 0.001; **p < 0.01; *p < 0.05.

Mice were ovariectomized to remove endogenous ovarian steroids at day 0 (d0), then administered estradiol (E2, Sigma-Aldrich, Poole) by subcutaneous injection daily on d7-9 (5ug.kg^-1^ in 200ul sesame oil) and on d13-15 (0.25ug.kg^-1^ in 200ul sesame oil) with a P4 pellet inserted on d13. On day 15 a decidualization stimulus was administered by intrauterine injection of 20ul sesame oil into the uterus *via* transvaginal delivery using a non-surgical embryo transfer device (NSET). Animals were sacrificed 4 days later and uterine tissues collected (**Figure 1A**, (23)). The decidualization response was immediately scored in each horn as outlined in **Figure 1B**. Specifically a non-decidualized horn had no decidualization reaction whatsoever (Non), whereas a fully decidualized horn had continuous decidualization along the majority (>50%) of its length (Full). Partial decidualization is any level of decidualization between these two extremes (**Figure 1B**). Collectively, samples designated Full or Partial are referred to as decidualized (Dec).

Finasteride (Sigma-Aldrich, Poole) was dissolved in 100% EtOH, then diluted to 5% v/v in sesame oil to a final concentration of 12.5mg.mL^-1^ and 100uL (50 mg.kg^-1^) was administered *via* intraperitoneal injection daily from the point of the decidualization stimulus onwards, i.e. d15-18, as show in **Figure S1A**. Finasteride is a synthetic 4-azasteroid that competitively inhibits both type I and II 5α-reductase enzymes in rodents (24, 25).

DHT (Sigma-Aldrich, Poole) was dissolved in 100% ethanol (EtOH) then diluted to 5% v/v in 0.4% w/v methylcellulose solution for a final DHT concentration of 2mg.mL^-1^. 100uL (8mg.kg^-1^) was injected subcutaneously on d15 at the point of decidualization stimulus. We have previously shown that this dose is sufficient to elicit changes in endometrial tissues (10, 26). At the end of experiments, mice were terminated by exposure to rising concentrations of CO_2_ combined with cervical dislocation.

### Histology and immunolabeling

Uterine tissue for histology was fixed in 4% paraformaldehyde then dehydrated and embedded in paraffin blocks and sectioned according to standard protocols. Protocols for 3,3’-diaminobenzidine (DAB) and immunofluorescence staining were performed as previously reported (10, 27). Briefly, slides were dewaxed, rehydrated, and subjected to antigen retrieval in pH 6.0 citrate buffer using a microwave. Following a wash in phosphate buffered saline (PBS) slides were incubated with 3% H_2_O_2_ solution in PBS for 15min to remove endogenous peroxidase activity. Following several PBS washes, slides were incubated with normal goat serum solution (NGS, PBS containing 20% goat serum and 0.05% (w/v) bovine serum albumin) to block non-specific interactions. Anti-CD31 primary antibody (Abcam, ab28364, 1:500 dilution) in NGS solution was added and incubated overnight at 4°C. Following several washes, slides were incubated with goat-raised peroxidase-conjugated secondary antibody (Abcam, ab7171, 1:500 dilution) for 1hr at room temperature. Following several washes, slides were incubated with either DAB reagent or Opal red 570 reagent for 10 minutes or until sufficiently developed. After a brief wash, slides were counterstained either with hematoxylin and eosin as per standard procedures, or with DAPI before being mounted with coverslips.

Slides were imaged using a Zeiss Z1 microscope, or a Zeiss AxioScan Slide Scanner using conventional fluorescent setups.

### Vessel quantification

To quantify vessels, regions of non-decidualized endometrium (Non) or decidua (Dec samples) were selected digitally on 20X magnification slide scans of CD31-labelled uterus sections and overlaid with a grid with edge length 250 pixels (~ 28μm). The intersections on the grid were sequentially classified as either vessel or not vessel, and the total proportion of vessel to non-vessel intersections was used as a measure of vessel count. Additionally, each time a vessel was identified, it was given an irregularity score equaling the number of intersections on a tree used to trace out the vessel’s cross-sectional shape. For example, a circular or ‘L’ shaped cross-section would have a score of 0, a ‘T’ shape a score of 1, an ‘H’ shape a score of 2, etc. Irregularity scores were averaged across all observations.

### RNA extraction

Uterine samples for RNA analysis were collected in RNALater solution (Qiagen, Hilden, Germany) and stored at -80°C until tissue extraction. For decidualized samples, only regions of uterus containing decidualized endometrium were selected. Samples were thawed and homogenized in 1 mL Trizol reagent using a TissueLyser machine (6min, 25Hz). Homogenate was transferred to MaXtract High Density tubes along with 200ul chloroform and 100ul RNase free water, shaken vigorously and centrifuged. The aqueous layer was added to RNeasy spin columns (Qiagen, Hilden, Germany) and processed as per manufacturer’s instructions.

### Quantitative RT-PCR

cDNA was synthesized from 100ng.uL^-1^ RNA using Superscript VILO cDNA synthesis kit (Thermo Fisher Scientific Life Sciences, Schwerte, Germany) as per the manufacturer’s instructions. Thermal cycler settings were 25°C for 10min, 42°C for 60min, 85°C for 5min. Primers were designed using Universal Probe Library Assay Design Center (Roche Applied Science, Burgess Hill, UK) or, when this was discontinued, using Neoformit (https://primers.neoformit.com/Accessed: 6/4/22; sequences in **Supplementary Data 1**). Primers were synthesized by Eurofins MWG Operon (Ebersberg, Gemany). Reactions were run in duplicate on a Quantstudio 5 384-well PCR machine (Thermo Fisher Scientific Life Sciences, Schwerte, Germany) using the following settings: 95°C for 10min then 40 cycles of 95°C for 15s and 60°C for 1min. A serial dilution of a standard cDNA solution (consisting of a mix of all samples tested) was used to plot a standard curve and expression levels interpolated from this. Data was processed using QuantStudio Design and Analysis Software.

### Nanostring gene expression analysis and data processing

Samples were analyzed on the NanoString nCounter Analysis System using a NanoString Mouse Pancancer Pathways Panel kit (Nanostring, Edinburgh, UK). RNA at 20ng.uL^-1^ was used. Processing was performed by the Host and Tumour Profiling Unit Microarray Services, Institute of Genetics and Cancer, University of Edinburgh, as per the manufacturer’s instructions. Briefly, the reporter codeset was mixed with hybridization buffer and added to each of the RNA samples, followed by the capture codeset. This mix was hybridized at 65°C for 18hr. Following hybridization samples were loaded into the provided cartridges on the nCounter prep station and processed using the High Sensitivity protocol. Cartridges were then sealed and read using the digital analyzer on the max setting. No QC flags were registered for any of the samples.

Data was processed using the nSolver analysis software. Data was normalized using a combination of positive controls and housekeeping genes. Firstly, background was subtracted using a background value of the mean plus two standard deviations of the negative control values. Normalization using positive controls and housekeeper genes was performed using the geometric mean to compute the normalization factor, and the manufacturer-provided list of housekeeper genes was used excluding the following genes based on the results of the geNorm algorithm in the Nanostring Advanced Analysis modules: *Hprt*, *Alas1*, *G6pdx*, *Gusb*, *Ppia*.

Normalized counts data was then analyzed for differential gene expression using R, specifically the edgeR analysis workflow. Dispersions were estimated with the *estimateDisp()* command and fit to the design model using *glmQLFit()*, before testing for differential expression using *glmQLFTest()*. Graphs were produced using the ggplot2 package. Finally, to perform gene ontogeny (GO) analysis we utilized the clusterProfiler analysis workflow. Ensembl IDs were mapped from the Bioconductor org.Mm.eg.db genome annotation and enrichment assessed using the *enrichGO()* command with the ‘universe’ set as the genes included in the Nanostring Pancancer Pathways panel. Graphs were produced using the *dotplot ()* and *cnetplot ()* commands from the enrichplot R package.

### Comparison of Nanostring gene expression with scRNAseq gene expression

Normalized counts and differential gene expression data was further analyzed in the context of publicly available single cell RNA sequencing (scRNAseq) data, accessed from NCBI’s Gene Expression Omnibus (28) through GEO Series accession numbers GSE160772 and GSE198556. These scRNAseq data defined the transcriptomic profile of over 20,000 perivascular, fibroblast and epithelial cell types present in the mouse uterus. The AverageExpression() command (Seurat) was used to calculate the mean expression of Nanostring DEgenes in perivascular, fibroblast and epithelial cell clusters from scRNAseq data. Gene expression matrices were normalized and clustered heatmaps produced using the pheatmap package. Expression values shown are scaled but not centered. This comparison allowed us to attribute the expression of genes that were found to be differentially expressed between Srd5a1^-/-^ and WT genotypes to certain cell fractions in the mouse endometrium.

### Creation and imaging of resin casts

Resin casts were prepared using a method previously validated in male mice (29). Briefly, female mice (Non and Dec) were culled using a terminal dose of sodium pentobarbital (150 mg/kg, ip) and perfusion fixation of the vasculature was achieved *via* the left ventricle. Heparinized PBS (heparin, 20 U/mL) was infused at 6 mL/min for 2 minutes. Low-viscosity resin (10 mL; Microfil MV-122; Flow Tech Inc) was prepared according to the manufacturer’s instructions and then infused *via* the left ventricle. Uterine tissues were recovered, trimmed, fixed in paraformaldehyde and embedded in 1.5% low melting point agarose (Invitrogen, UK), dehydrated in methanol (100%; 24 hours) and optically cleared in benzyl alcohol:benzyl benzoate (1∶2 v/v; 24 hours). Imaging was carried out as described in Rebourcet et al. with a positive control tissue being a previously prepared mouse testis (29).

### Evans Blue assay of vascular permeability

Evans Blue (EB) dye binds to albumin in the blood so its presence in tissues, which can be quantified spectrophotometrically, implies a breakdown in the endothelial barrier. The Evans Blue dye (Sigma-Aldrich, Poole) was dissolved in sterile saline solution to a concentration of 0.5% w/v, and 200uL was injected i.v. (*via* tail vein) 30-90 minutes prior to cull *via* cervical dislocation. The interval between EB injection and cull was recorded and found not to associate with dye retention in tissues.

To quantify the amount of Evans blue in the tissues, following sacrifice a portion of the uterus was taken, precisely weighed, and transferred to 500μl of formamide. The gross appearance of the tissue was recorded using a digital camera. For decidualized samples, only sections of uterus containing decidualized endometrium were selected. Samples were incubated for 24h at 55°C to extract the dye, then tissue pieces were discarded, the solution centrifuged to remove debris, and absorbance read in triplicate at 610nm.

### Data processing and statistical analysis

Unless otherwise stated, data was processed using Microsoft Excel and Python. Statistics were performed using either Python or Graphpad Prism 5.

In the analysis of Nanostring data, two-way ANOVAs were performed on all genes found to be expressed and ANOVA results corrected for multiple comparisons using the Benjamini and Hochberg method (30). The false discovery rate (FDR) was 0.05.

**Figure 2:**
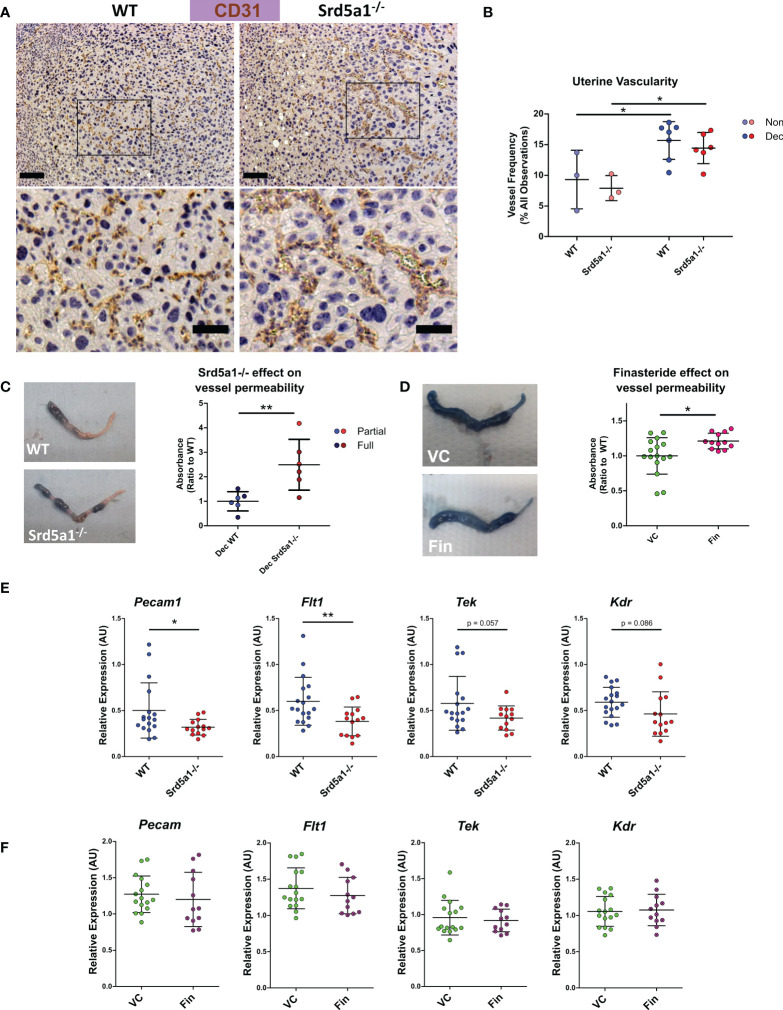
Evidence of a vascular phenotype in Srd5a1^-/-^ mice following induction of decidualization. **(A)** Representative images of CD31^+^ cells in WT and Srd5a1^-/-^ decidua. Zoomed-in views are shown from regions indicated by boxes. Scale bars are 100μm and 50μm respectively. **(B)** Quantification of vessel density in in WT and Srd5a1^-/-^ non-decidualized endometrium and decidua. **(C, D)** Images of decidualized uterine horns following intravenous injection of Evans Blue and respective quantification of absorbance in uterine tissue extracts in decidualized horns of WT and Srd5a1^-/-^ **(C)** or vehicle control (VC) and finasteride (Fin) treated mice **(D)**. **(E, F)**. Expression of angiogenic genes *via* qPCR in decidualized tissue (partial and full) from WT and Srd5a1^-^/- mice **(E)**, or VC and Fin mice **(F)**. Plots in B analyzed by two-way ANOVA with Bonferroni *post-hoc tests;* **(C–F)** analyzed by two-tailed t test. **p < 0.01; *p < 0.05.

In the analysis of Evans Blue absorbance data, absorbance readings are shown as a ratio relative to the mean value of WT or vehicle-treated WT data. In the case of data from **Figure 2**, ratios have been calculated separately for partially and fully decidualized horns and the results combined for the subsequent statistical analysis.

Statistical tests performed for other comparisons are stated in figure legends and the text.

## Results

### 5α-reductase deficiency impairs decidualization in mouse uterus

To investigate the functional requirement for 5α-reductase during decidualization we induced decidualization in WT and Srd5a1^-/-^ mice using a previously validated protocol (**Figure 1A**; (23)). We used a ternary system to classify the extent of decidualization. Uterine horns were classified as non-, partially- or fully-decidualized (Non, Partial, Full, respectively; **Figure 1B**). For the purposes of analysis individual uterine horns were treated as separate biological entities. In instances where only the decidualized regions of the uterus are analyzed Partial and Full horns were grouped together: referred to as ‘decidualized’ (Dec). Decidualization was associated with a significant increase in *Srd5a1* mRNA expression in wildtype mice which was absent in knockout mice (**Supplementary Figure 1**). Decidualization was observed in both genotypes, however Srd5a1^-/-^ uteri had a noticeably impaired decidualization response compared with those of WT. Specifically uteri from Srd5a1^-/-^ mice had fewer ‘Full’ horns and decidualized areas in ‘Partial’ horns were smaller (**Figure 1C**). When quantified and compared statistically we were able to reject the hypothesis that the number in each of the decidualization response groups is the same between genotypes, which supports our interpretation that decidualization is impaired in Srd5a1^-/-^ mice (**Figure 1D**).

In line with expectations, uterine weight increased significantly in Dec horns compared to non-decidualized horns in both WT and Srd5a1^-/-^ mice (**Figure 1E**). Uterine horn weights of partially decidualized horns from WT mice were significantly heavier than those from Srd5a1^-/-^ females (**Figure 1F**). The expression of mRNAs encoded by *Prl8a2*, a marker of decidualization, was significantly increased in Dec compared to Non horns in WT mice. A similar, non-significant, trend for increased *Prl8a2* mRNA expression was observed in Srd5a1^-/-^ mice (**Figure 1G**).

To complement these findings we tested the impact of transiently blocking 5α-reductase activity in WT mice using the pharmacologic 5α-reductase inhibitor finasteride (for 4 days from days 15-18; **Supplementary Figure 2A**). Notably this treatment regime did not significantly affect the decidualization response, uterine horn weights, nor expression of *Prl8a2* (**Supplementary Figures 2B, C, D**). Thus transient inhibition of 5α-reductase under this paradigm was insufficient to replicate the effects of the global *Srd5a1* knock-out on decidualization responses.

### 5α-reductase is required for normal endothelial barrier function and vascular remodeling during decidualization

To investigate if impaired decidualization in Srd5a1^-/-^ mice was also associated with deficient vascular morphology and function we stained blood vessels with the endothelial cell marker CD31. Observational analysis of tissue sections suggested blood vessels in mutant mice appeared less numerous and more dilated compared to WT (**Figure 2A**). To quantify the vessels, counts were performed on samples fluorescently labelled for CD31 (**Supplementary Figures 3A**). This analysis revealed a significant increase in vessel frequency in decidualized compared to non-decidualized uterine horns (**Figure 2B**). The mean vessel frequency in Srd5a1^-/-^ decidualized uteri was lower than in WT but no statistically significant difference was detected (**Figure 2B**). Vessel irregularity was also quantified, revealing a significant increase with decidualization, but no distinct effect of genotype (**Supplementary Figures 3B**).

We attempted to map the three-dimensional architecture of vessels using the resin cast method, however accurate casts could not be obtained which we believe is due to the high permeability of vessels in the decidualized tissues (**Supplementary Figures 4**). This finding and observations from histological slides of dilated vessels in the decidual tissue of Srd5a1^-/-^ mice led us to consider whether vessel permeability might be altered as a consequence of ablation of the gene in mice.

To quantify whether these morphological changes lead to functional deficiency in endothelial barrier integrity, we investigated vascular permeability using Evans Blue assay. We observed a significant increase in permeability of decidualized uterine horns from Srd5a1^-/-^ compared to WT mice (**Figure 2C**). In mice treated with finasteride, similar trends were observed, and there was a significant increase in permeability in decidualized horns following finasteride treatment (**Figure 2D**).

### Changes in angiogenic gene expression during decidualization are attenuated in Srd5a1^-/-^ mice

To investigate the potential molecular basis for the vascular phenotype observed in Srd5a1^-/-^ uteri, we quantified the expression of key angiogenic genes in decidualized horns from WT and Srd5a1^-/-^ mice. A significant decrease in the expression of *Pecam1* and *Flt1* was detected in Srd5a1^-/-^ uteri compared to WT (**Figure 2E**). The mean expression of *Tek* (TIE2/Angiopoietin-1 receptor) and *Kdr* (VEGFR2) was also decreased but this was not statistically significant (**Figure 2E**). When gene expression was analyzed in WT mice treated with finasteride there was no detectable change in the expression of the same panel of angiogenic genes (**Figure 2F**).

### 5α-reductase deficiency attenuates decidualization-induced changes in gene expression

**Figure 3:**
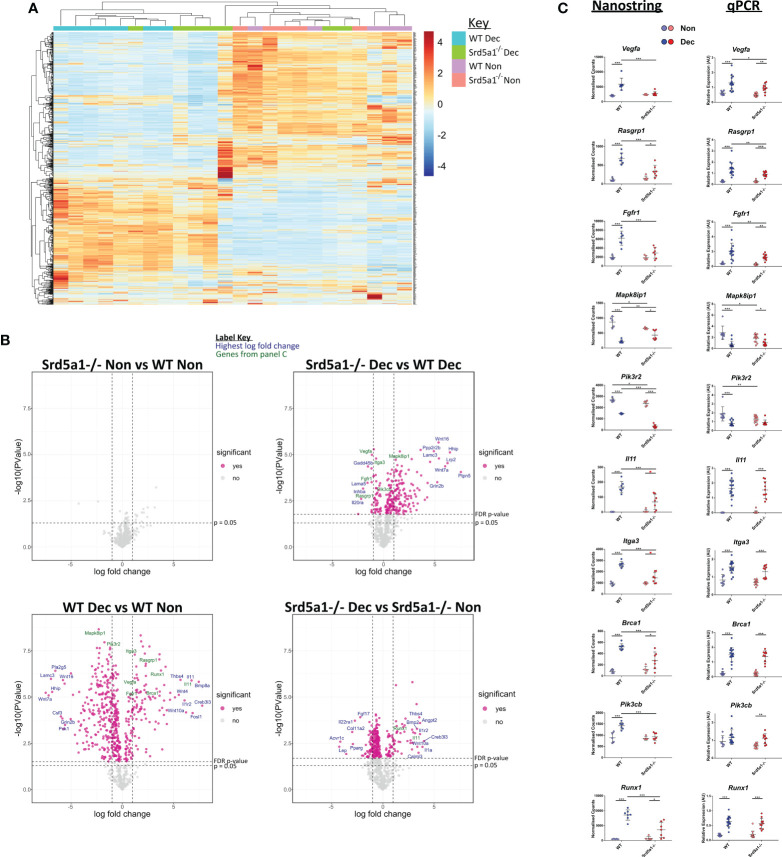
Effect of 5α-reductase deficiency on global gene expression during decidualization. **(A)** Unsupervised clustering and heatmap of WT and Srd5a1^-/-^ decidualized and non-decidualized tissue RNA analyzed with Nanostring Pancancer Pathways gene expression panel. The major gene expression differences occur with decidualization. **(B)** Volcano plots for the pairwise comparisons between experimental groups, as indicated. Significantly differentially expressed genes at a false discovery rate (FDR) of 0.05 are colored pink. **(C)** Selection of genes changing significantly with either genotype, or interaction of genotype and decidualization, by two-way ANOVA (FDR = 0.05). Count values in the Nanostring data and respective validation by qPCR in groups with additional samples is shown. Plots in C analyzed by two-way ANOVA with Bonferroni *post-hoc* tests. *p < 0.05; **p < 0.01; ***p < 0.001.

To discover genes and signaling pathways that are altered by deletion of *Srd5a1* in decidualized endometrium we analyzed the transcriptome of Non and Dec uterine tissue from WT and Srd5a1^-/-^ mice using the Nanostring Pancancer Pathways panel. To visualize the general patterns of gene expression, count values were subjected to unsupervised hierarchical clustering and visualized on a heat map (**Figure 3A**). Clustering of samples forms two major arms, one containing exclusively Dec samples and the other mostly Non. These findings are consistent with decidualization being a major driver of changes in gene expression in these samples. Notably, within the Dec arm Srd5a1^-/-^ samples largely clustered together and closer to the Non arm, and several decidualized Srd5a1^-/-^ samples are found within the Non arm, suggesting a transcriptional phenotype in Srd5a1^-/-^ Dec uterus which was not the same as in the WT Dec tissue.

To interrogate the differential expression of genes between the two genotypes, we performed four ratio comparisons: Srd5a1^-/-^ Non *vs.* WT Non; Srd5a1^-/-^ Dec *vs.* WT Dec; WT Dec *vs.* WT Non; and Srd5a1^-/-^ Dec *vs.* Srd5a1^-/-^ Non (**Figure 3B**). This analysis revealed no significantly differentially expressed (DE) genes between genotypes in non-decidualized tissue (Srd5a1^-/-^ Non *vs.* WT Non), suggesting a minimal role for 5α-reductase in regulating uterine gene expression in the absence of decidualization. In stark contrast, decidualization in WT mice induced significant changes in the expression of many genes: 507 or 66% of the total analyzed (WT Dec *vs.* WT Non; **Table 1**). Interestingly, the majority (71%) of the DE genes in this panel were down-regulated by decidualization. In comparison with the WT data, gene expression changes in Srd5a1^-/-^ decidualized tissues (Srd5a1^-/-^ Dec *vs.* Srd5a1^-/-^ Non) were greatly suppressed, with fewer DE genes (299, 39%) and smaller changes in expression level for both up- and down-regulated genes (**Table 1**). When the expression of genes in decidualized uteri of WT and Srd5a1^-/-^ mice was directly compared (Srd5a1^-/-^ Dec *vs.* WT Dec), many significantly DE genes are observed (254, 33%), most of which are up-regulated in Srd5a1^-/-^ compared to WT (80%). Notably when significantly DE genes were considered 95% of the up-regulated genes in this comparison are downregulated in the WT Dec *vs.* WT Non comparison.

**Table 1:** Summary of differentially expressed genes using ratio analysis and 2-Way ANOVA analysis of Pancancer Pathways Nanostring panel.

Ratio Analysis	Differentially expressed genes (DE)			log_2_FC of DE genes		
	(#)	(% total)	Upregulated (% DE)	Downregulated(Mean ± 95% CI)	Upregulated(Mean ± 95% CI)	Absolute(Mean ± 95% CI)
Srd5a1^-/-^ Non *Vs.* WT Non	0	0	NA	NA	NA	NA
Srd5a1^-/-^ Dec *Vs.* WT Dec	254	33.0	79.5	-0.84 (-0.71–0.97)	1.56 (1.39–1.74)	1.41 (1.27–1.56)
WT Dec *Vs.* WT Non	507	65.8	29.0	-1.68 (-1.54–1.82)	1.94 (1.67–2.20)	1.75 (1.63–1.88)
Srd5a1^-/-^ Dec *Vs.* Srd5a1^-/-^ Non	299	38.8	25.4	-0.84 (-0.76–0.91)	1.51 (1.27–1.76)	1.01 (0.92–1.10)
**2-Way ANOVA Analysis**						
Decidualization	464	61.9				
Genotype	27	3.6				
Interaction	33	4.4				

Non, non-decidualized uterus; Dec, decidualized uterus; DE, differentially expressed; FC, fold-change; CI, confidence interval.

NA = Not applicable.

To identify genes differentially regulated by the Srd5a1^-/-^ genotype, we performed two-way ANOVA on Nanostring data using the count number of all genes and the variables decidualization (Non/Dec) and genotype (WT/Srd5a1^-/-^). Results are shown in **Table 1**. We identified 47 genes significantly regulated by either genotype or the interaction of genotype and decidualization, and gene lists can be found in **Supplementary Data 2**. We further validated key differentially expressed genes from this set by qPCR in a cohort containing additional samples and these tissues showed gene expression patterns that matched those seen in the Nanostring counts or followed similar trends (**Figure 3C**).

### *In silico* analysis of gene networks associated with defective decidualization in Srd5a1^-/-^ mice identifies deficient expression of angiogenesisregulatory pathways

To gain insight into which gene expression pathways are driving decidualization, and how they are affected by deletion of *Srd5a1*, we performed gene ontology (GO) analysis based on DE genes identified from our Nanostring data analysis. Upregulated and downregulated genes from WT Dec *vs.* WT non and Srd5a1^-/-^ Dec *vs.* WT Dec comparisons were analyzed separately.

No significant GO terms were identified based on genes upregulated by decidualization (WT Dec *vs.* WT Non), however in the downregulated genes there were several terms related to transmembrane transporter activity (**Supplementary Figures 5A**). Category netplot (cnet) analysis of the enriched genes showed that all the GO terms were related to the same set of genes (**Supplementary Figures 5B**). Many of these genes were subunits of the L-type voltage-dependent Ca^2+^ channel (VDCC) which has been shown to be downregulated with decidualization in endometrial stromal cells (31). In agreement with these findings, many significant GO terms were identified for genes upregulated in Srd5a1^-/-^ decidua compared to WT (Srd5a1^-/-^ Dec *Vs.* WT Dec) all of which related to transmembrane ion transport (**Supplementary Figures 5C, D**).

**Figure 4:**
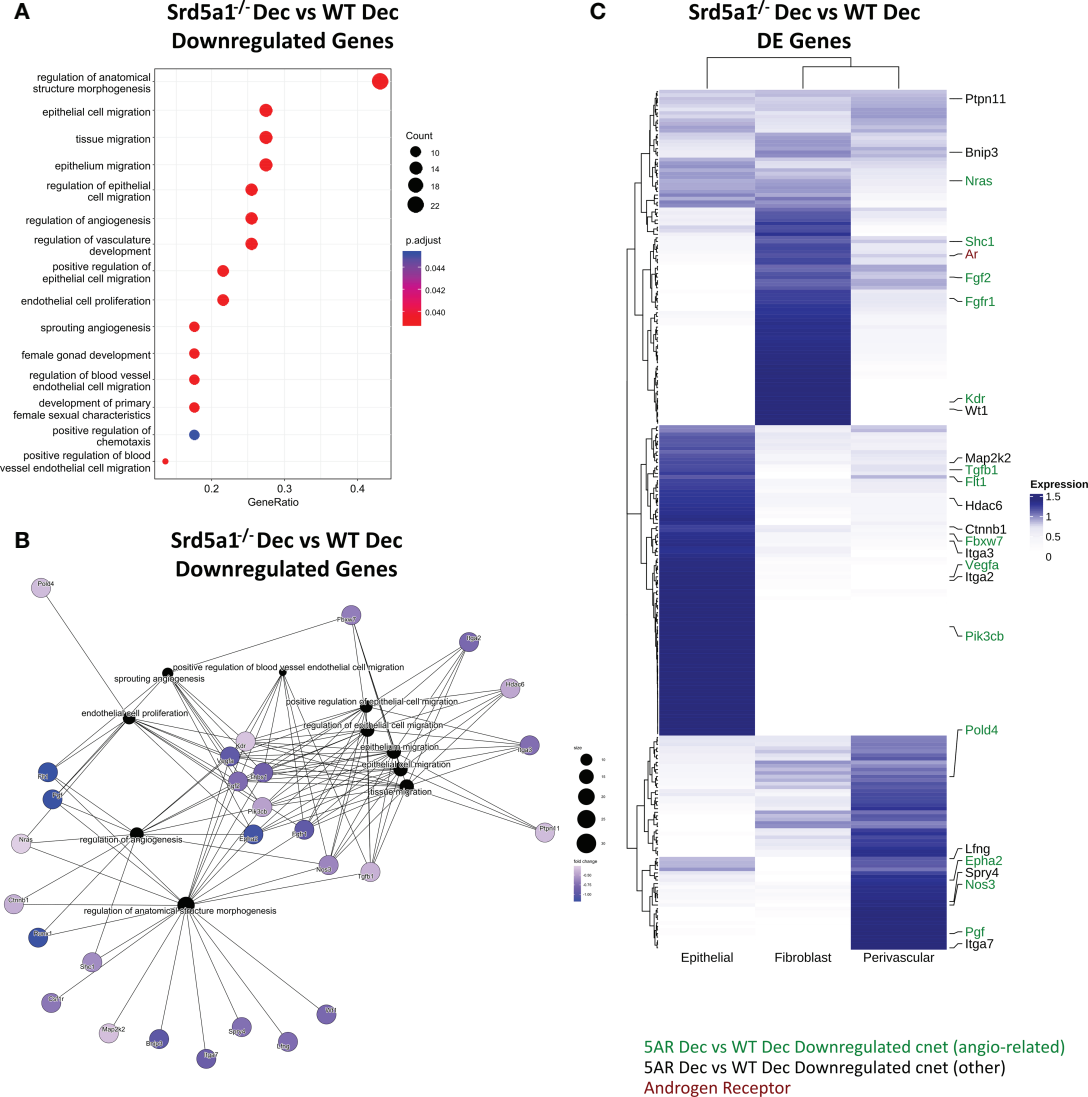
Gene networks associated with defective decidualization in Srd5a1^-/-^ mice. **(A)** Biological process gene ontogeny (GO) of genes significantly downregulated in Srd5a1^-/-^ *vs.* WT decidualized uterine horns. All significant terms shown. **(B)** Category netplot (cnet) embedding indicating genes shared between GO terms for genes significantly downregulated in Srd5a1^-/-^ *vs.* WT decidualized uterine horns. **(C)** Heatmap showing expression of top DE genes from the Srd5a1^-/-^ compared to WT decidualized uterine horns and their relative abundance in uterine cell subsets; epithelial, fibroblast and perivascular, as derived from annotation in previously published scRNAseq dataset of mouse uteri. Genes appearing in the cnet plot in B are labelled and color coded based on whether they link to angiogenesis-related GO terms (green) or not (black).

Significant GO terms were also identified for genes downregulated in Srd5a1^-/-^ decidualized uterus compared to WT (Srd5a1^-/-^ Dec *Vs.* WT Dec; **Figure 4A**). The majority related to cell migration, however there was also a large proportion of GO terms related to the regulation of the vasculature, endothelial cells, or angiogenesis. Cnet analysis highlighted multiple highly connected genes from canonical angiogenesis pathways such as the VEGF pathway (**Figure 4B**; *Flt1* (VEGFR1), *Kdr* (VEGFR2), *Vegfa*, *Pgf*). This Cnet also shows that many of the genes related to migration are also linked to angiogenesis pathways. This analysis highlights that gene regulatory pathways that promote angiogenesis are significantly downregulated in decidualized uteri from Srd5a1^-/-^ mice.

### Genes differentially regulated by 5α-reductase deficiency are expressed by stromal and epithelial cell populations in cycling uterus

To identify which cells in the uterus were likely to have altered gene expression as a result of deletion of *Srd5a1*, we compared the results of our Nanostring analysis with our previously published single cell RNA sequencing (scRNAseq) data from cycling mouse uterus which included mesenchymal cells (i.e. fibroblasts and perivascular cells), which are AR^+^ (10, 32), and epithelial cells (27). Expression of significantly DE genes from the Srd5a1^-/-^ Dec *vs.* WT Dec ratio analysis were sorted by hierarchical clustering (**Figure 4C**). All genes had detectable expression in at least one cell population and AR was expressed in stromal (fibroblast>perivascular), but not epithelial, populations. Together, stromal fibroblasts and perivascular cells have detectable expression of the majority of the angiogenic and migratory genes whose expression is regulated by 5α-reductase deficiency identified in the cnet plot (**Figure 4B**). Perivascular cells play a key role in supporting the growth, stability, and integrity of blood vessels, as well as playing a major role in angiogenesis. This analysis highlights AR^+^ perivascular cells and fibroblasts as plausible cell types mediating the angiogenesis related effects of SRD5A1 activity.

### DHT rescues impaired decidualization response in Srd5a1^-/-^ mice

**Figure 5:**
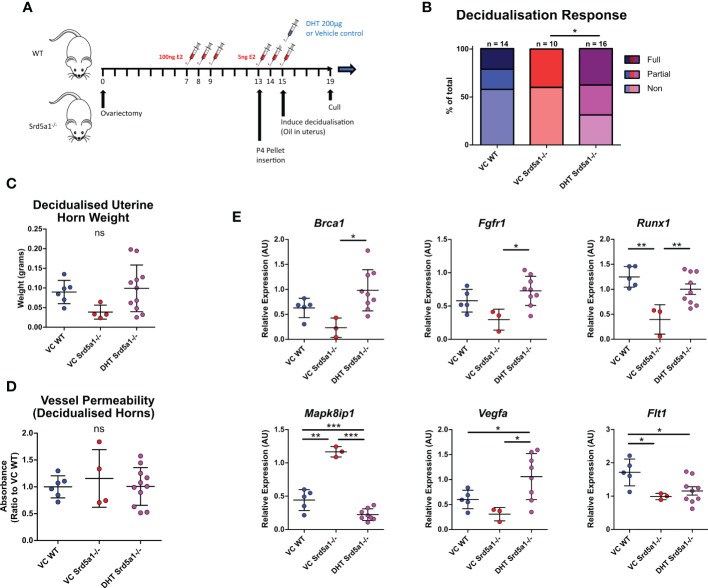
Impact of DHT administration on decidualization response in Srd5a1^-/-^ mice. **(A)** Schema of decidualization induction experiment in WT and Srd5a1^-/-^ mice with addition of DHT or vehicle control (VC) at point of decidualization induction. **(B)** Contingency table depicting proportion of horn responses in WT and Srd5a1^-/-^ mice treated with DHT or VC. **(C)** Wet weight of uterine horns. **(D)** Quantification of Evans Blue dye absorbance in uterine tissue extracts. **(E)** Expression of selected genes analyzed by qPCR. Plot in B analyzed by chi-squared test; C,D,E analyzed by one-way ANOVA and Neuman-Keuls *post-hoc* tests. *p < 0.05; **p < 0.01; ***p < 0.001; ns = no significant differences detected.

Given that AR+ cell types were associated with differential gene expression in Srd5a1^-/-^ mice, we postulated that a lack of intracrine androgen may have altered cell function during decidualization in the presence of 5α-reductase deficiency. To test whether impaired decidualization in Srd5a1^-/-^ mice is due to a reduction in intracrine/paracrine DHT, we performed a rescue experiment by administering exogenous DHT coincident with the decidualization stimulus (**Figure 5A**). Following this treatment, the rate of decidualization in DHT-treated mutants (Srd5a1^-/–^DHT) significantly increased compared to vehicle-treated mutants (Srd5a1^-/–^VC) and was statistically indistinguishable from vehicle-treated WT animals (WT-VC; **Figure 5B**). Furthermore, the weight of decidualized uterine horns was also the same between WT-VC and Srd5a1^-/–^DHT groups (**Figure 5C**).

We also tested whether the vessel permeability phenotype seen in Srd5a1^-/-^ animals could be rescued by exogenous DHT and found that Evans Blue dye detection in extracts from decidualized Srd5a1^-/–^DHT was equivalent to that from WT-VC horns, demonstrating that the vascular permeability phenotype is also rescued by DHT administration (**Figure 5D**).

Finally, we tested whether altered gene expression identified in the Srd5a1^-/-^ uterus was affected by administration of DHT. Notably, DHT restored mRNA expression levels to WT-VC levels (*Brca1*, *Fgfr1*, *Mapk8ip1*, *Runx1*, *Vegfa*; **Figure 5E**), and others showed the same trend (*Flt1*, *Pecam*, *Il11*, *Rasgrp1*; **Figure 5E**, **Supplementary Figure 6A**). For some mRNAs there was no evidence of a rescue (*Itga3*, *Pik3cb*, *Pik3r2*; **Supplementary Figure 6B**). The most significant change in gene expression in response to DHT supplementation occurred in genes related to angiogenesis signaling and downstream signal transduction pathways such as *Vegfa*, *Fgfr1*, and *Mapk8ip1*, suggesting that these stromal/vascular associated pathways are androgen-regulated during decidualization.

Overall this shows that gross tissue changes and many of the transcriptomic changes associated with impaired decidualization in Srd5a1^-/-^ mice can be restored by administration of DHT.

## Discussion

Correct and timely decidualization is essential to the establishment of pregnancy, and defects in decidualization are linked with implantation failure, pregnancy complications and menstrual disorders such as heavy bleeding (4, 5, 17). We and others have shown that a correct androgen balance is required for optimal decidualization and fertility (11, 33–35), but the exact nature of this requirement, e.g. through direct androgen signaling or by acting as precursors to other steroids, and its downstream effects have not been established in an *in vivo* setting. Identifying the mechanisms through which androgens support the establishment of pregnancy is therefore essential to our understanding of endometrial function and to the development of future fertility treatments. To this end, we have utilized a mouse line and pharmacological approaches to disrupt steroid 5α-reductase activity, which is necessary for the production of DHT, in the endometrium during a model of decidualization and analyzed the effect of this intervention on uterine physiology, transcriptome, and capacity to decidualize.

These investigations have revealed that androgen signaling in the form of locally produced DHT is required for decidualization to proceed as normal. We have shown that decidualization is severely impaired in the absence of SRD5A1, the enzyme responsible for local DHT production, and that exogenous DHT administration rescues this effect. Previously, we have shown *in vitro* that hESF upregulate androgen biosynthetic enzymes and produce androgens as they are induced to decidualize, and that AR antagonism blocks intracrine signaling and delays the full differentiation of these cells (12). The current findings extend this paradigm, demonstrating that intracrine androgen signals are required *in vivo* to drive decidualization and promote vascular remodeling.

It is possible that lower decidualization rates observed in Srd5a1^-/-^ mice represent a delay in tissue remodeling compared to WT. In the context of the tightly regulated fertility cycles of mammals such a delay would be sufficient to severely affect fertility. This observation aligns with the reduced fecundity of Srd5a1^-/-^ mice (22), which may in part be due to decidualization/implantation defects.

Gene expression changes and the results of the Evans Blue permeability assay suggest a strong role for DHT signaling in vascular remodeling during decidualization. Decidualized tissue of Srd5a1^-/-^ mice had downregulated expression (*vs.* WT) of gene pathways from two major areas: cell migration and angiogenesis/vascular genes. The process of angiogenesis is inseparable from migration, and migratory genes have previously been shown to be androgen-dependent *in vitro* in endometrial stromal cells (11). It is well established that activated AR promotes, indirectly, VEGF expression and elements of the VEGF pathway (11, 36–38); and components of this pathway, such as *Flt1* and *Vegfa*, were substantially downregulated in Srd5a1^-/-^ Dec uteri compared to WT. We propose that the vascular phenotypes and impaired decidualization shown here in Srd5a1^-/-^ mice are a direct result of impaired VEGF and other angiogenic signaling due to a lack of promotion from AR. This paradigm opens up new avenues of exploration for treatments of decidualization disorders involving impaired androgen signaling as a contributing factor, such as premature ovarian failure, adrenal insufficiency, or in women of advanced maternal age (39).

In our study, finasteride administration did not affect the rate of decidualization or uterine horn weight in WT mice whereas in Srd5a1^-/-^ mice DHT rescued these features. There are several possible explanations for this discrepancy. The simplest is that, since finasteride was only administered at the point of decidualization stimulus, sufficient residual DHT was already present in the tissue to support a normal decidualization response (40, 41). Although finasteride inhibits both type I and II 5α-reductase enzymes in rodents (24, 25) some residual SRD5A1 activity will persist in the presence of finasteride (42) and this may also be sufficient to support decidualization. Furthermore, it is possible that global deletion of *Srd5a1* throughout development and the lifespan of these mutant mice has broader impacts on uterine function that cannot be recapitulated by transient pharmacologic inhibition of the enzyme. Finasteride did however increase vessel permeability during decidualization in WT mice, similar to the phenotype observed in Srd5a1-/- mice. These findings may represent distinct responses to intracrine androgens such that AR-positive perivascular cells are more sensitive to inhibition of 5α-reductase than stromal fibroblasts during decidualization and/or that the impact of inhibition on these different cell types is time-dependent. It is notable that endothelial nitric oxide synthase (Nos3), which is a potent, acute mediator of endothelial function (43), is downregulated during decidualization in Srd5a1-/- mice. This suggests that pathways downstream of intracrine androgens may be acutely or temporally regulated and thus differentially impacted by transient inhibition *via* finasteride.

A limitation of this study is that targeting the SRD5A1 protein is likely to have effects on steroid hormone signaling beyond its elimination of DHT. In previous work with the same mouse line, the cause of its defective parturition phenotype was identified as a lack of another 5α-reduced androgen, 5α-androstan-13α,17β-diol, indicating there may be specific effects of other 5α-reduced steroids that are not accounted for here (44). A separate mechanism by which the Srd5a1^-/-^ mutation may affect results is through diversion of precursor steroids towards other pathways, as occurs during pregnancy when a mid-gestational T surge is aromatized rather than 5α-reduced, causing fetal death due to estrogen excess in Srd5a1^-/-^ mice (21). These factors may account for why some gene expression changes were not rescued by DHT administration, and provide an alternative explanation for why finasteride administration does not fully recapitulate the Srd5a1^-/-^ phenotype.

Androgen availability, particularly DHEA production, declines with age and there is a concomitant age-related increase in risk of adverse pregnancy outcomes (45, 46). Although much of this risk is related to oocyte quality (47), our study suggests potential for endometrial function to be affected by age which may be particularly relevant in conditions such as recurrent implantation failure where endometrial responses are impaired (48). The current study demonstrates the importance of intracrine androgen signaling to early pregnancy remodeling, including decidualization and vascular adaptation for pregnancy. Given that intracrine androgen production is directly and positively correlated to precursor (DHEA) availability (18), a decrease or deficiency in DHEA could impair decidualization capacity. Further studies are therefore warranted to investigate a possible causal link between androgen deficiency, endometrial function and disorders of pregnancy. Future work in response to this study should investigate intracrine androgen signaling effects in the context of natural pregnancy, for example testing whether DHT can improve fecundity in Srd5a1^-/-^ female mice. Although administration of androgens *in utero* could affect fetal development (49), the paradigm in this study highlights opportunities for pre-pregnancy androgen modulation to support decidualization. Furthermore, potential treatment approaches could use inactive precursors such as DHEA to mitigate direct androgenizing effects; an approach we have previously used to enhance decidualization responses *in vitro* (18). It will also be valuable to ascertain the exact mechanism through which AR signaling activates the VEGF pathway, and whether stimulating this pathway can likewise improve fertility in the context of androgen depletion or deficiency. This will facilitate an improved diversity of treatment options for cases where androgen supplementation is not desirable (39).

In summary, using a well-validated mouse model we have confirmed that intracrine androgen signaling, stimulated by locally produced DHT, is required for the robust and timely execution of pathways that stimulate gene expression and changes in cell function essential for a robust decidualization response. By furthering our understanding of the role androgens play in endometrial receptivity and related endometrial disorders these findings will aid in the development of fertility treatments and other interventions.

## Data availability statement

The original contributions presented in the study are included in the article/**Supplementary material**. Further inquiries can be directed to the corresponding author.

## Ethics statement

The animal study was reviewed and approved by University of Edinburgh Animal Welfare and Ethical Review Board and in accordance with the Animals (Scientific Procedures) Act 1986 under UK law.

## Author contributions

IS designed and performed the experiments, analyzed the data, interpreted the results, and wrote the manuscript. PK performed the experiments, analyzed the data, and interpreted the results. DR, RA, FC, and LS performed the experiments. DL provided study materials. PS conceived the study, designed the experiments, interpreted the results and revised the manuscript. DG conceived the study, designed and performed the experiments, analyzed the data, interpreted the results, and revised the manuscript. All authors contributed to the article and approved the submitted version.

## Funding

MRC programme grants to PS (G1100356/1 and MR/N024524/1); MRC Programme Grant to LS (MR/N002970/1); Wellcome Trust Fellowship to DG (220656/Z/20/Z); Scottish Funding Council Research Adaption Fund to IS; importation of the mice from Jackson laboratories was paid from Wellcome Trust grant 072217/Z/03/Z.

## Acknowledgments

We thank Prof. Ruth Andrew (University of Edinburgh) for founder stocks of Srd5a1-/- mice. We are grateful to Dr Alison Munro and the Host and Tumour Profiling Unit (Cancer Research UK – Edinburgh Centre) for support for Nanostring analysis, Dr Pamela Brown from Biomolecular core facility and facility staff from the Bioresearch and Veterinary Services team.

## Conflict of interest

The authors declare that the research was conducted in the absence of any commercial or financial relationships that could be construed as a potential conflict of interest.

## Publisher’s note

All claims expressed in this article are solely those of the authors and do not necessarily represent those of their affiliated organizations, or those of the publisher, the editors and the reviewers. Any product that may be evaluated in this article, or claim that may be made by its manufacturer, is not guaranteed or endorsed by the publisher.
